# Untargeted metabolite profiling on the water-soluble metabolites of edible bird’s nest through liquid chromatography-mass spectrometry

**DOI:** 10.14202/vetworld.2020.304-316

**Published:** 2020-02-17

**Authors:** Shi-Ruo Tong, Ting-Hun Lee, Soon-Keng Cheong, Yang-Mooi Lim

**Affiliations:** 1 Department of Pre-clinical Sciences, Faculty of Medicine and Health Sciences, Universiti Tunku Abdul Rahman, Jalan Sungai Long, Bandar Sungai Long, 43000, Kajang, Cheras, Selangor, Malaysia; 2 Department of Bioprocess and Polymer Engineering, School of Chemical and Energy Engineering, Universiti Teknologi Malaysia, 81310, Johor Bahru, Johor, Malaysia; 3 Centre for Cancer Research, Faculty of Medicine and Health Sciences, Universiti Tunku Abdul Rahman, Jalan Sungai Long, Bandar Sungai Long, 43000, Kajang, Cheras, Selangor, Malaysia

**Keywords:** edible bird’s nest, extraction method, liquid chromatography-mass spectrometry, untargeted metabolite profiling

## Abstract

**Background and Aim:**

Edible bird’s nest (EBN) is the nutrient-rich salivary bioproduct produced by swiftlets in Southeast Asia. Currently, researchers are exploring the therapeutic effects of EBN, such as cell growth promotion, antioxidant content, antiviral effects, bone strengthening, eyes care, and neuroprotection bioactivities. The therapeutic effects of EBN have been studied through different extraction methods but the metabolites profile of the EBN in each extract has not yet been elucidated. This study aimed to profile the water-soluble metabolites of EBN prepared in different extraction methods. Subsequently, an extraction method will be selected as an ideal extraction method for untargeted metabolite profiling on the water-soluble metabolites in EBN.

**Materials and Methods:**

In this study, water-soluble metabolites of EBN extracted by the four extraction methods were subjected to metabolite profiling through liquid chromatography-mass spectrometry (LC-MS). The extraction methods were acid extraction(ABN), pancreatic extraction (EzBN), eHMG extraction, and spray drying of HMG extraction (pHMG). The metabolite profiles, such as the number of metabolites and their identities in each extraction method, were evaluated through LC-MS analysis.

**Results:**

The identity of metabolites present in the four extraction methods is inconsistent. Based on LC-MS analysis, only one and six metabolites were extracted differently through EzBN and ABN, respectively, in the first pre-screening. Through the second LC-MS screening on pHMG and eHMG extraction methods, eHMG was selected as an ideal extraction method due to the highest numbers of water-soluble metabolites with an amount of 193 was detected. Besides, eHMG extraction method was able to extract sialic acid and a high percentage of secondary metabolites.

**Conclusion:**

This study suggests that eHMG is the ideal extraction method for extracting higher number of water-soluble metabolites from EBN and could be further developed as an extraction method for industry application. In addition, this study also has identified the types of primary and secondary metabolites present in EBN.

## Introduction

Edible bird’s nest (EBN) is a well-known bioproduct made from the saliva secretion of swiftlet, specifically from the two genera of *Aerodramus* and *Collocalia*. The swiftlet from the two genera is mostly habitat in Southeast Asia [1,2]. The main constituents of EBN are proteins, carbohydrates, lipids, and a group of minerals such as calcium, sodium, potassium, magnesium, phosphorus, iron, zinc, copper, chromium, and selenium [2-5]. EBN has been regarded as traditional Chinese medicine by the practitioners in Qing dynasty due to its recuperative properties [1,6]. The recuperative properties of EBN are highlighted with the effect of boosting immune system, treating malnutrition, improving metabolism, enhancing skin complexion and alleviating asthma, helping in phlegm clearance, relieving cough, nourishing children, libido raising, enhancing renal function, recovery from illness and surgery, as well as improving concentration [7]. Recently, EBN is further demonstrated for its properties on suppressing the virus, inflammation and oxidative stress, strengthening bone, eye caring, and neuroprotective properties [8-14]. On the other hand, Roh *et al*. [15] and Kong *et al*. [16] have reported the proliferative effects of EBN on human adipose-derived stem cells and normal human fibroblasts with the presence of epidermal growth factor-like activity. In summary, EBN acts as a dual function bioproduct with both its nutritional and therapeutic values.

To study the constituents of EBN and its therapeutic effects, the development of an ideal extraction methodology of EBN is very important. Several extraction methodologies were developed and used for studying the bioactivities of EBN. The study by Guo *et al*. [9] documented strong inhibition of influenza viruses by EBN extract that is pre-treated with pancreatin. Besides, Abidin *et al*. [11] also reported that the EBN extract prepared by eHMG extraction method successfully stimulated and enhanced the proliferation of corneal keratocytes in wound healing without altering their functionality. Chua *et al*. [17] prepared EBN extracts by the water extraction method (HMG). These extracts exhibited strong chondroprotective effects on osteoarthritis (OA). In addition, Aswir and Wan Nazaimoon [18] have documented acid-extracted EBN exhibited an anti-inflammation effect by significantly reducing the production of the inflammatory protein, tumor necrosis factor-alpha. In view of all the works, it is observed that different EBN extract obtained through different extraction methods showed different therapeutic effects. One possible explanation is because the extraction of an active component is highly dependent on the extraction method employed. Thus far, the identity of the metabolites in each of these extractions has not yet been further studied for the underlying mechanism of actions for their therapeutic effects. Hence, future study could be carried out to confirm the therapeutic effects of the metabolites.

Metabolite profiling is a powerful scientific tool for a complete investigation of a group of small molecules. This approach often used in analyzing biological components for the identification of potential biomarkers for certain diseases [19]. Recently, metabolite profiling has gained fame in food classification [20,21]; this is due to its untargeted analysis approach with the potential to cover the whole or the maximum metabolomics molecular information of foods. One of the examples of using the metabolite profiling approach on EBN has successfully demonstrated in the study done by Chua *et al*. [22]. The metabolites of the EBN were extracted through the chloroform/methanol solvent extraction, which was then successfully identified through gas chromatography-mass spectrometry (MS) and liquid chromatography-MS (LC-MS) techniques.

Since water is commonly used to prepare EBN essence for consumption and the metabolites of EBN are not fully established yet, this study aimed to preliminary profile the water-soluble metabolites of EBN prepared in different extraction methods. Subsequently, an extraction method will be selected as an ideal extraction method for untargeted metabolite profiling on the water-soluble metabolites in EBN.

## Materials and Methods

### Ethical approval

The study did not involve any live animals, so no ethical approval was required.

### Chemicals

LC-MS grade formic acid and acetonitrile were purchased from Fisher Scientific (Waltham, MA, USA). Deionized water was obtained from a Barnstead GenPure water purification system (Thermo Fisher Scientific Inc., Waltham, MA, USA).

### Sample collection, preparation, and extraction

Raw unclean EBNs samples were collected collectively from different swiftlet premises located in Johor, Malaysia. The feathers and impurities were manually removed with forceps, and the raw unclean EBN was ground with mortar and pestle. Ground EBN was sieved through a 0.4 mm wire mesh to further separate the smaller pieces of feathers and impurities. The unclean EBN powder was then placed in an air force oven at 50-55°C overnight to reduce the moisture content.

There were four extraction methods selected for the comparison in this study, namely, eHMG, pHMG, ABN, and EzBN extraction methods. The raw unclean EBN was extracted with the proprietary methods of eHMG [11] and pHMG (the spray-dried of HMG extract) [17] that were innovated and standardized by School of Chemical and Energy Engineering in Universiti Teknologi Malaysia (UTM). These methods were modified based on the methods presented by Oda *et al*. [23] and Goh *et al*. [24]. Besides, another acid extraction (ABN) and pancreatin extraction (EzBN) were developed by the team of Universiti Tunku Abdul Rahman (UTAR) in 2016 [25] with some modification from the methods presented by Aswir and Wan Nazaimoon [18] and Goh *et al*. [9].

#### eHMG and pHMG

Due to the proprietary issue on these two extraction methods, the details of these two methods were unable to be described in this report.

#### Acid extraction (ABN)

The EBN powder was suspended in deionized water at 0.2% (w/v) and left for 24 h. The mixture was then boiled at 80°C with 2% (v/v) of 0.4 M sulfuric acid for 4 h. The extract was allowed to cool down and centrifuged at 2716 g (5000 rpm) for 15 min. The pH of the supernatant collected was neutralized to pH 7.0. The white precipitated formed was removed through centrifugation with 2716 g (5000 rpm) for 15 min at 4°C. The supernatant was collected and kept at 4°C for further analysis.

#### Pancreatin extraction (EzBN)

The EBN powder was suspended in deionized water at 0.2% (w/v) and left for 24 h. The EBN mixture was boiled at 100°C for 30 min. An amount of 1 ml of 0.5 mg/ml pancreatin was added into EBN mixture and was allowed for the reaction at 45°C for 4 h with pH 8.5-9.0. The enzyme was inactivated by heating at 90°C for 10 min. The supernatant was collected after centrifugation at 2716 g (5000 rpm) for 15 min. The extract was kept at 4°C.

Before subjecting the extracts to LC-MS analysis, all the four extracts were centrifuged at 9660 g (12,000 rpm) for 10 min and the supernatant of the extracts was filtered through 0.2 µm polytetrafluoroethylene membranes.

### Quadrupole time-of-flight (QTOF) LC-MS analysis

The four EBN extracts were qualitatively analyzed using Agilent 6560 Ion Mobility QTOF (IM-QTOF) LC-MS system that coupled with the Agilent 1290 ultra-high-performance liquid chromatography (Agilent Technologies, USA). The metabolites present in the EBN extracts were separated through POROSHELL 120 EC-C18 (4.6×100 mm; 2.7 μ; Agilent Technologies, USA) chromatographic column with the mobile phase that consisted of (A) 0.1% formic acid in water and (B) 0.1% formic acid in acetonitrile. All the four EBN extracts were undergone the first pre-screening evaluation with the elution of 5-95% B (0.0-1.0 min) and 95-5% B (1.0-15.0 min). The flow rate was set at 1.0 ml/min. The two extracts with a higher number of metabolites were selected and further subjected for the second LC-MS screening with modified mobile phase elution. The condition of the modified elution was set as follows: 5% B (0.0-2.0 min), 5-15% B (2.0-4.0 min), 15-25% B (4.0-6.0 min), 25-35% B (6.0-8.0 min), 35-45% B (8.0-10.0 min), 45-50% B (10.0-12.0 min), 50-75% B (12.0-16.0 min), 75-100% B (16.0-20.0 min), 100-5% B (20.0-20.1 min), and isocratic at 5% (20.1-25 min). The flow rate was modified to 0.3 ml/min.

The other setting parameters for IM-TOF analysis remained the same throughout the analysis process. The injection volume was 1 µl and the column temperature was maintained at 40°C. The acquisition of the metabolites was performed in positive (ES+) mode. The mass spectra were recorded over an m/z range from 100 to 1000. Deionized water was used as the background blank. Whereas, the operating conditions of the mass spectrometer were set as follows: Capillary voltage of 4000 V, nozzle voltage of 500 V, and fragmentor voltage of 365 V were maintained. Nebulizer pressure (N_2_) was kept at 20 psi, drying gas temperature was maintained at 225°C. Drying gas flow was 13 L/min and sheath gas flow was 12 L/min at 400°C.

### Data mining and metabolites identification

The metabolite features from the acquired MS spectral raw data were extracted with the untargeted molecular feature extraction algorithm in Agilent MassHunter Workstation - Qualitative Analysis software B.07.00 (Agilent Technologies, USA). The algorithm filtered off the peak height with 100 counts to avoid the noise spectral picking, as well as the mass of internal reference ions with 121.0967 and 922.1389. Then, the algorithm locates the covariant ions in the chromatogram and grouped them as a single metabolite feature using the information of mass, isotopic distribution with common organic elements (C, H, O, N, P, Cl, F, and S), charge-state and adducts of sodium, potassium, and ammonium. The extracted metabolite features were characterized by retention time (RT) and intensity.

The identity of the extracted metabolite features was searched against METLIN Personal Metabolite Database in the MassHunter software based on the accurate mass and RT (optional). The mass and RT tolerance of the compound identity matching was restricted to ±5 ppm and ±0.1 min (optional), respectively. The accuracy of the identity of each metabolite was calculated as a score. The metabolites list of each extract was retained if the identity of the metabolite fulfilled the threshold score of 80, and the error of database matching was less than ±5 ppm.

## Results and Discussion

### The efficiency of EBN extraction methods

The method of extraction is a crucial process that maximizes the extraction of the bioactive metabolites from EBN. To search for an ideal extraction method for the untargeted metabolite profiling of EBN, four different extraction methods with the therapeutic effects were assessed and evaluated. For example, pancreatin extraction with antiviral effect as reported by Guo *et al*. [9]; eHMG extraction with the effect of enhancing proliferation of corneal keratocytes by Abidin *et al*. [11]; HMG extraction showed chondroprotective effect on OA as documented by Chua *et al*. [17]; and finally the acid extraction with anti-inflammation bioactivities reported by Aswir and Wan Nazaimoon [18]. The approach of LC-MS is recognized with its high sensitivity, accuracy, and reproducibility [26-28]; thus, there was no technical replicate done in this untargeted metabolite profiling analysis.

The number of detected metabolites in each of the extraction method was analyzed by MassHunter software. Nearly 37-67% out of the total metabolites from the four different extracts were putatively identified by matching with the METLIN metabolites database. The complete information of all the identified metabolites in each extraction method is detailed in Table-1. The identities of the extracted metabolites are unique among the four different extracts, suggesting that there is no single extraction method that could extract all types of metabolites due to the differences in natural physicochemical properties of the metabolites [29-32].

**Table-1:** Information of the metabolites in each extracts with first pre-screening by QTOF LC-MS.

Number	RT (min)	Ion	Mass	m/z	Molecular formula	Score	DB differences (ppm)	Putatively identified metabolites

**ABN**								
1	1.001	(M+NH_4_)^+^	104.0375	122.0713	C_6_H_4_N_2_	85.65	−0.48	4-Cyanopyridine
2	1.005	(M+H)^+^	273.1082	274.1154	C_9_H_15_N_5_O_5_	97.11	−3.06	4a-Peroxy-tetrahydrobiopterin
3	1.005	(M+H)^+^	291.1206	292.1278	C_14_H_17_N_3_O_4_	89.12	4.47	Serinyl-Tryptophan
4	1.005	(M+H)^+^	309.1332	310.1406	C_17_H_18_F_3_NO	87.45	2.62	Fluoxetine
5	1.039	(M+Na)^+^	325.0796	348.0687	C_14_H_15_NO_8_	84.49	0.49	Pancratistatin
6	1.083	(M+Na)^+^	291.0943	314.0835	C_11_H_17_NO_8_	95.46	3.83	2-Deoxy-2,3-dehydro-N-acetylneuraminic acid

**EzBN**								

1	1.502	(M+H)^+^	109.0643	110.0715	C_5_H_7_N_3_	85.25	−2.46	2-Aminomethylpyrimidine

**eHMG**								

1	0.741	(M+H)^+^	379.1125	380.1197	C_14_H_21_NO_11_	83.05	−2.62	Chondroitin
2	0.924	(M+Na)^+^	333.1523	356.1415	C_13_H_23_N_3_O_7_	80.80	3.82	Ser Asp Leu
3	1.007	(M+H)^+^	385.2081	386.2152	C_15_H_27_N_7_O_5_	83.05	−1.87	Asn Pro Arg
4	1.042	(M+H)^+^	311.1692	312.1764	C_12_H_21_N_7_O_3_	91.60	4.34	Arginyl-Histidine
5	1.112	(M+H)^+^	344.2172	345.2244	C_14_H_28_N_6_O_4_	84.88	0.07	Gly Ile Arg
6	1.138	(M+NH_4_)^+^	384.2144	402.2484	C_20_H_32_O_7_	81.25	1.10	Cinnzeylanol
7	1.311	(M+H)^+^	387.2238	388.2313	C_15_H_29_N_7_O_5_	94.39	−2.03	Arg Asn Val
8	1.383	(M+H)^+^	373.2333	374.2405	C_16_H_31_N_5_O_5_	81.27	−2.19	Lys Asn Leu
9	4.299	(M+H)^+^	654.3986	655.4060	C_35_H_58_O_11_	93.38	−1.00	Filipin III
10	4.316	(M+H)^+^	130.0741	131.0813	C_5_H_10_N_2_O_2_	87.43	1.25	L-cis-3-Amino-2-pyrrolidinecarboxylic acid
11	4.342	(M+H)^+^	114.0433	115.0505	C_4_H_6_N_2_O_2_	86.61	−3.03	Muscimol

**pHMG**								

1	1.019	(M+H)^+^	166.0270	167.0344	C_8_H_6_O_4_	85.71	−2.59	3-Formylsalicylic acid
2	1.020	(M+Na)^+^	383.1430	406.1321	C_14_H_25_NO_11_	98.38	−0.64	Lacto-N-biose I
3	1.022	(M+Na)^+^	309.1065	332.0956	C_11_H_19_NO_9_	82.57	−1.76	N-Acetyl-b-neuraminic acid
4	1.023	(M+Na)^+^	325.0792	348.0685	C_14_H_15_NO_8_	82.22	1.85	Pancratistatin
5	1.024	(M+H)^+^	203.0798	204.0870	C_8_ H_13_NO_5_	86.39	−1.97	N2-Acetyl-L-aminoadipate
6	1.026	(M+Na)^+^	291.0960	314.0851	C_12_H_13_N_5_O_4_	84.24	2.72	Toyocamycin
7	1.075	(M+Na)^+^	291.0954	314.0847	C_11_H_17_NO_8_	83.71	0.12	2,7-Anhydro-alpha-N-acetylneuraminic acid
8	1.314	(M+H)^+^	137.0478	138.0551	C_7_H_7_NO_2_	87.40	−1.17	2-Pyridylacetic acid
9	1.319	(M+Na)^+^	145.0770	168.0660	C_9_H_9_N_2_	81.66	−2.63	4-Aminomethylindole
10	1.402	(M+H)^+^	245.1385	246.1457	C_10_H_19_N_3_O_4_	81.33	−3.66	Asn Leu
11	1.514	(M+H)^+^	135.0544	136.0617	C_5_H_5_N_5_	84.12	0.94	Adenine
12	1.542	(M+NH_4_)^+^	256.0582	274.0920	C_11_H_12_O_7_	93.89	0.53	Piscidic Acid
13	5.692	(M+H)^+^	101.0840	102.0912	C_5_H1_1_NO	87.59	0.67	2-Methylpropanal O-methyloxime

RT=Retention time, DB=Database, LC-MS=Liquid chromatography-mass spectrometry, QTOF=Quadrupole time-of-flight

**Figure-1:**
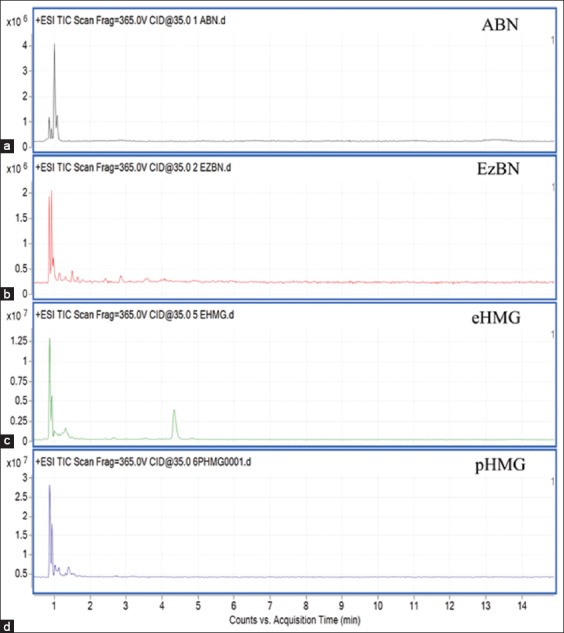
Total ion chromatograms of the first liquid chromatography/mass spectrometry (LC-MS) pre-screening on edible bird’s nest extraction methods (a) ABN, (b) EzBN, (c) eHMG, and (d) pHMG. The LC-MS chromatograms are obtained from ES+mode.

Based on the mobile phase for compound separation in the first screening evaluation, there were significant differences in the number of extracted metabolites under each extraction method (Table-2a). The highest total number of metabolites obtained was from pHMG extract and followed by eHMG extract. The total number of metabolites detected in both of pHMG and eHMG extracts was greater than EzBN and ABN extracts, with approximately 20-30 times and 4-5 times, respectively. However, the LC-MS separation for each extract was not well defined by referring to the chromatograms obtained (Figure-1). Therefore, the second screening evaluation was carried out with an improved LC-MS mobile phase. Both of the eHMG and pHMG extraction methods were selected to undergo the second screening evaluation since they showed greater efficacy in extracting the higher number of metabolites from EBN in the first screening evaluation.

**Table-2:** Number of metabolites detected and identified by QTOF LC-MS in each of the extracts for the first prescreening and the second screening with the optimized LC-MS parameters.

Extracts	Total metabolites	Putatively identified metabolites	Metabolites after filtering*
(a) First pre-screening			
ABN	18	7	6
EzBN	3	2	1
eHMG**	69	26	11
pHMG**	85	34	13
(b) Second screening with optimized parameters			
eHMG	775	468	193
pHMG	168	96	42

* Metabolites filtering is based on the presence of contaminants, the score and database matching error (ppm).

** The extracts were selected for the second screening with the optimized LC-MS parameters. LC-MS=Liquid chromatography-mass spectrometry, QTOF=Quadrupole time-of-flight

**Figure-2:**
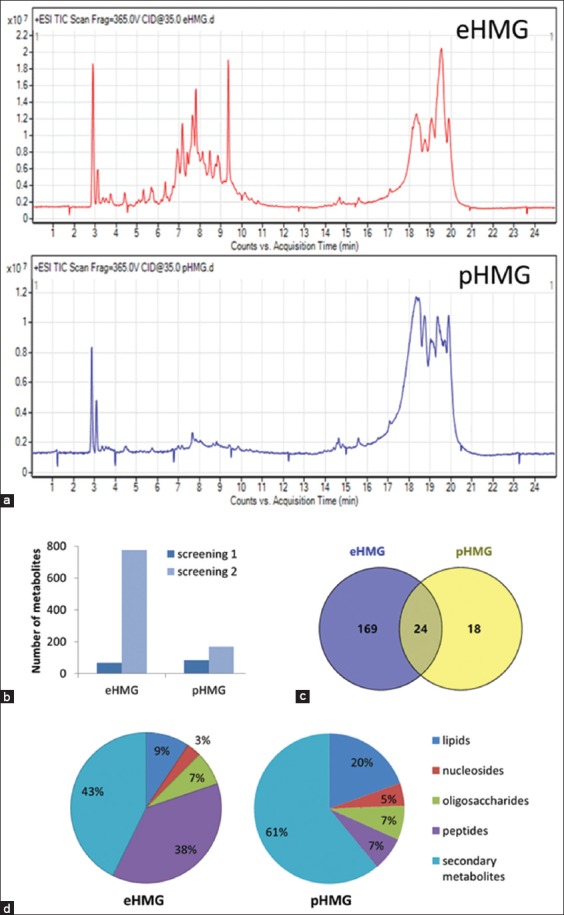
The second liquid chromatography/mass spectrometry (LC-MS) screening with optimized parameters on eHMG and pHMG extracts. (a) Total ion chromatograms of eHMG and pHMG extraction methods selected from the first pre-screening. The LC-MS chromatograms were obtained from ES+mode. (b) The efficiency comparison between the first and second screening for both eHMG and pHMG extracts. (c) The number of metabolites that found similar between pHMG and eHMG extracts. The comparison was made based on the identified metabolites that the contaminant was filtered off. (d) The classification of edible bird’s nest metabolites in eHMG and pHMG extracts. The classification was based on the metabolite identities after removing the contaminants.

The second screening evaluation with an optimized LC-MS mobile phase for separating compounds has greatly improved the elution efficacy and increased the number of analyzed metabolites (Figure-2a and b). The good separation in the liquid chromatography has broadened the range of eluted metabolites. Hence, the second screening evaluation has provided a better comparison between the eHMG and pHMG extraction methods. The eHMG extraction method has successfully recovered a significant number in total extracted metabolites as compared with pHMG (Table-2b). There were 193 metabolites detected from eHMG extraction method (Table-2b), which are more than 26 non-polar metabolites detected in the study done by Chua *et al*. [22]. Therefore, the eHMG extraction method was selected as the ideal extraction method because it provided the maximal recovery of the number of water-soluble metabolites present in EBN.

### The metabolite profile of extraction methods

In the second screening evaluation, there were approximately more than half out of the total metabolites (60.39% and 57.14% of metabolites, respectively) from eHMG and pHMG extracts that were putatively identified. The information of the retained metabolites for both eHMG and pHMG extraction methods in the second screening evaluation are shown in Tables-3 and 4, respectively. Based on the comparison between eHMG and pHMG extraction methods in the second screening evaluation, 24 out of the total identified metabolites were found to be similar in each extract (Figure-2c). The result indicated that the eHMG extraction method not only extracted a greater number of metabolites but also there were approximately 57.14% of the metabolites from pHMG extraction method which were found to be similar to eHMG. The identities of the metabolites that found to be similar in both of the extraction methods are marked in Tables-3 and 4.

**Table-3:** Information of the metabolites in eHMG extract. The metabolites are identified by QTOF LC-MS with second screening evaluation.

Number	RT (min)	Ion	Mass	m/z	Molecular formula	Score (DB)	DB differences (ppm)	Putatively identified metabolites
1	7.678	(M+NH_4_)^+^	188.1052	206.1388	C_9_H_16_O_4_	87.23	−1.78	(+/−)-Ethyl 3-acetoxy-2-methylbutyrate
2	6.351	(M+H)^+^	156.0533	157.0605	C_6_H_8_N_2_O_3_	86.21	1.11	(S)-3-(Imidazol-5-yl)lactate
3	9.285	(M+NH_4_)^+^	146.0481	164.0819	C_8_H_6_N_2_O	87.68	−0.82	1(2H)-Phthalazinone
4	7.814	(M+H)^+^	225.1118	226.1190	C_10_H_15_N_3_O_3_	85.52	−1.99	1-(Methylnitrosoamino)-4-(3-pyridinyl)-1,4-butanediol
5	7.154	(M+NH_4_)^+^	151.1003	169.1341	C_9_H_13_NO	85.35	−3.75	1,2,3,4,5,6-Hexahydro-5-methyl-7H-cyclopenta[b]pyridin-7-one
6	19.916	(M+H)^+^	310.2875	311.2947	C_20_H_38_O_2_	82.62	−1.10	15Z-eicosenoic acid
7	19.852	(M+NH_4_)^+^	168.1882	186.2220	C_12_H_24_	93.33	−2.37	1-Dodecene*
8	17.086	(M+H)^+^	203.0811	204.0882	C_9_H_17_NS_2_	90.55	−4.19	1-Isothiocyanato-7-(methylthio)heptane*
9	13.104	(M+H)^+^	115.0456	116.0528	C_5_H_9_NS	95.32	−0.49	1-Isothiocyanatobutane*
10	19.913	(M+NH_4_)^+^	392.4382	410.4720	C_28_ H_56_	95.74	0.02	1-Octacosene*
11	8.497	(M+Na)^+^	303.1824	326.1717	C_18_H_25_NO_3_	93.54	3.33	1-O-Desmethyltetrabenazine
12	6.266	(M+H)^+^	129.0429	130.0503	C_5_H_7_NO_3_	94.98	−2.57	1-Pyrroline-4-hydroxy-2-carboxylate
13	7.633	(M+H)^+^	365.1324	366.1397	C_14_H_23_NO_10_	98.61	−0.58	2-(acetylamino)-1,5-anhydro-2-deoxy-4-O-b-D-galactopyranosyl-D-arabino-Hex-1-enitol
14	15.590	(M+H)^+^	171.1087	172.1160	C_9_H_17_NS	90.62	−3.11	2,5-Dihydro-4,5-dimethyl-2-(1-methylpropyl)thiazole*
15	7.631	(M+H)^+^	291.0958	292.1030	C_11_H_17_NO_8_	98.24	−1.31	2,7-Anhydro-alpha-N-acetylneuraminic acid
16	8.822	(M+NH_4_)^+^	418.1835	436.2172	C_19_H_30_O_10_	98.30	1.05	2-[4-(3-Hydroxypropyl)-2-methoxyphenoxy]-1,3-propanediol 1-glucoside
17	8.685	(M+H)^+^	113.0843	114.0917	C_6_H_11_NO	86.02	−2.34	2-Acetylpyrrolidine*
18	5.701	(M+NH_4_)^+^	155.0950	173.1288	C_8_H_13_NO_2_	86.86	−2.51	2-Amino-2-Norbornanecarboxylic acid
19	5.702	(M+H)^+^	190.0958	191.1029	C_7_H_14_N_2_O_4_	90.69	−2.50	2-Amino-4-[(2-hydroxy-1-oxopropyl)amino]butanoic acid
20	7.725	(M+NH_4_)^+^	239.1067	257.1405	C_14_H_13_N_3_O	81.23	−3.70	2-amino-a-phenyl-1H-Benzimidazole-5-methanol
21	10.087	(M+NH_4_)^+^	94.0785	112.1124	C_7_H_10_	86.34	−2.46	2-Methyl-1,3-cyclohexadiene
22	7.103	(M+H)^+^	155.0701	156.0774	C_6_H_9_N_3_O_2_	84.30	−4.09	3-(Pyrazol-1-yl)-L-alanine
23	6.949	(M+NH_4_)^+^	118.0421	136.0759	C_8_H_6_O	87.41	−1.96	3,5,7-Octatriyn-1-ol
24	7.656	(M+NH_4_)^+^	477.1901	495.2239	C_26_H_27_N_3_O_6_	93.78	−0.31	3,5-Pyridinedicarboxylic acid, 2,6-dimethyl-4-(3-nitrophenyl)-, methyl 2-[methyl(phenylmethyl)amino]
25	9.346	(M+H)^+^	186.1374	187.1449	C_9_H_18_N_2_O_2_	96.27	−3.33	3-[(3-Methylbutyl)nitrosoamino]-2-butanone
26	6.347	(M+NH_4_)^+^	129.0791	147.1129	C_6_H_11_NO_2_	99.35	−0.98	3-acetamidobutanal
27	7.395	(M+H)^+^	194.1061	195.1134	C_10_H_14_N_2_O_2_	85.27	−2.80	3-Hydroxy-N-glycyl-2,6-xylidine (3-Hydroxyglycinexylidide)
28	7.632	(M+H)^+^	196.0377	197.0449	C_9_H_8_O_5_	85.10	−2.44	3-Methoxy-4,5-methylenedioxybenzoic acid
29	19.921	(M+NH_4_)^+^	278.2972	296.3311	C_20_H_38_	85.63	0.46	3Z,6Z-Eicosadiene
30	10.783	(M+H)^+^	218.1424	219.1495	C_13_H_18_N_2_O	92.62	−2.42	4-[2-(Propylamino)ethyl]-1,3-dihydro-2H-indol-2-one
31	7.627	(M+Na)^+^	145.0766	168.0658	C_9_H_9_N_2_	86.63	0.02	4-Aminomethylindole
32	8.253	(M+NH_4_)^+^	153.1156	171.1493	C_9_H_15_NO	92.51	−1.46	4-Butyl-2,5-dimethyloxazole
33	5.641	(M+NH_4_)^+^	167.1316	185.1654	C_10_H_17_NO	85.56	−3.51	4-Butyl-2-ethyl-5-methyloxazole
34	10.175	(M+H)^+^	104.0373	105.0446	C_6_H_4_N_2_	87.29	1.25	4-Cyanopyridine
35	8.933	(M+H)^+^	466.2196	467.2267	C_19_H_30_D_3_N_3_O_8_S	87.39	−4.25	4-hydroxy Nonenal Glutathione-d3
36	6.351	(M+H)^+^	166.0379	167.0452	C_7_H_6_N_2_O_3_	83.68	−0.52	4-Hydroxy-3-nitrosobenzamide
37	8.457	(M+NH_4_)^+^	129.0430	147.0769	C_5_H_7_NO_3_	96.21	−2.91	4-Oxoproline*
38	6.327	(M+NH_4_)^+^	168.0903	186.1241	C_8_H_12_N_2_O_2_	86.78	−2.26	4-PIOL
39	8.928	(M+NH_4_)^+^	139.0638	157.0977	C_7_H_9_NO_2_	86.17	−3.48	5-Acetyl-2,4-dimethyloxazole
40	7.722	(M+NH_4_)^+^	267.1016	285.1354	C_15_H_13_N_3_O_2_	81.08	−3.24	5-benzyl-5-(pyridin-3-yl)imidazolidine-2,4-dione
41	7.815	(M+NH_4_)^+^	165.1159	183.1497	C_10_H_15_NO	98.24	−3.05	5-Methyl-2-(1-pyrrolidinyl)-2-cyclopenten-1-one*
42	7.400	(M+H)^+^	241.1067	242.1140	C_10_H_15_N_3_O_4_	86.34	−1.88	5-Methyldeoxycytidine
43	9.361	(M+Na)^+^	597.3489	620.3383	C_31_H_51_NO_10_	89.94	4.09	5-O-β-D-Mycaminosyltylonolide
44	7.658	(M+NH_4_)^+^	139.0999	157.1337	C_8_H_13_NO	92.23	−1.66	5-Pentyloxazole
45	7.406	(M+H)^+^	172.0851	173.0924	C_7_H_12_N_2_O_3_	97.82	−1.94	5-δ-Hydroxybutyl Hydantoin
46	8.150	(M+Na)^+^	585.2945	608.2836	C_33_H_39_N_5_O_5_	91.39	1.04	8’,10’- Dihydroxydihydroergotamine
47	6.927	(M+H)^+^	216.0755	217.0829	C_8_H_12_N_2_O_5_	95.52	−4.14	8-Hydroxyalanylclavam
48	7.919	(M+NH_4_)^+^	228.1364	246.1703	C_12_H_20_O_4_	86.63	−1.26	9,12-dioxo-dodecanoic acid
49	16.039	(M+NH_4_)^+^	250.2300	268.2639	C_17_H_30_O	86.18	−1.19	9S,10R-Epoxy-3Z, 6Z-octadecadiene*
50	19.926	(M+NH_4_)^+^	364.4068	382.4407	C_26_H_52_	99.78	0.22	9Z-Hexacosene*
51	6.756	(M+NH_4_)^+^	217.0856	235.1194	C_11_H_11_N_3_O_2_	86.25	−2.08	Acetylhydrazinopthalazinone
52	9.279	(M+NH_4_)^+^	245.1170	263.1508	C_13_H_15_N_3_O_2_	96.87	−2.15	Acetyltryptophanamide
53	4.538	(M+H)^+^	135.0551	136.0623	C_5_H_5_N_5_	84.51	−4.22	Adenine*
54	7.198	(M+H)^+^	243.1221	244.1298	C_10_H_17_N_3_O_4_	85.40	−0.95	Ala Gly Pro
55	6.934	(M+NH_4_)^+^	236.1165	254.1504	C_12_H_16_N_2_O_3_	97.31	−1.88	Alanyl-DL-Phenylalanine
56	3.734	(M+H)^+^	226.1074	227.1145	C_9_H_14_N_4_O_3_	87.75	−3.48	Alanyl-Histidine*
57	8.374	(M+H)^+^	232.1578	233.1652	C_14_H_20_N_2_O	85.03	−0.85	Albine
58	6.159	(M+NH_4_)^+^	141.0796	159.1134	C_7_H_11_NO_2_	85.48	−4.19	Arecaidine
59	7.686	(M+H)^+^	271.1650	272.1723	C_11_H_21_N_5_O_3_	86.06	−1.91	Arginyl-Proline
60	7.155	(M+H)^+^	279.1216	280.1289	C_13_H_17_N_3_O_4_	85.73	1.14	Asn Phe
61	9.361	(M+NH_4_)^+^	378.1906	396.2245	C_18_H_26_N_4_O_5_	99.55	−0.62	Asn Val Phe
62	5.759	(M+H)^+^	368.1330	369.1404	C_15_H_20_N_4_O_7_	84.38	0.41	Asn-Lys-OH
63	3.515	(M+Na)^+^	349.1122	372.1015	C_12_H_19_N_3_O_9_	98.31	−0.19	Asp Thr Asp
64	7.189	(M+Na)^+^	559.3144	582.3037	C_31_H_45_NO_8_	94.22	0.15	Auriculine
65	3.073	(M+NH_4_)^+^	173.0434	191.0772	C_5_H_7_N_3_O_4_	87.00	1.51	Azaserine
66	14.410	(M+NH_4_)^+^	290.1788	308.2126	C_20_H_22_N_2_	83.40	−1.75	Azatadine
67	4.411	(M+NH_4_)^+^	472.2283	490.2622	C_27_H_36_O_5_S	93.08	0.12	BAY-u9773
68	7.655	(M+NH_4_)^+^	165.0796	183.1135	C_9_H_11_NO_2_	85.21	−4.05	Benzocaine
69	9.276	(M+H)^+^	234.1483	235.1557	C_12_H_18_N_4_O	94.24	−1.09	Benzoylagmatine
70	8.382	(M+Na)^+^	365.1733	388.1628	C_21_H_23_N_3_O_3_	82.56	1.83	Brevianamide B
71	3.342	(M+H)^+^	109.0644	110.0717	C_5_H_7_N_3_	96.49	−4.13	Brunfelsamidine*
72	8.118	(M+H)^+^	643.3329	644.3407	C_35_H_49_NO_10_	80.90	4.29	Buprenorphine 3-O-glucuronide
73	7.511	(M+NH_4_)^+^	224.1165	242.1504	C_11_H_16_N_2_O_3_	84.08	−2.03	Butalbital
74	6.960	(M+H)^+^	212.1166	213.1240	C_10_H_16_N_2_O_3_	80.31	−2.26	Butethal
75	6.757	(M+H)^+^	278.1270	279.1342	C_14_H_18_N_2_O_4_	95.45	−1.14	Carboxy-PTIO
76	8.511	(M+H)^+^	607.3124	608.3200	C_31_H_41_N_7_O_6_	90.53	−0.90	Chymostatin
77	17.086	(M+H)^+^	127.1365	128.1437	C_8_H_17_N	86.60	−2.89	Coniine*
78	6.940	(M+H)^+^	176.0947	177.1028	C_10_H_12_N_2_O	82.06	1.45	Cotinine
79	9.360	(M+Na)^+^	203.1303	226.1195	C_13_H_17_NO	96.10	3.31	Crotamiton
80	9.356	(M+H)^+^	224.1892	225.1965	C_13_H_24_N_2_O	93.91	−1.45	Cuscohygrine
81	7.537	(M+H)^+^	244.1215	245.1287	C_14_H_16_N_2_O_2_	86.62	−1.11	Cyclo(L-Phe-L-Pro)
82	6.992	(M+H)^+^	227.0914	228.0986	C_9_H_13_N_3_O_4_	95.88	−3.67	Deoxycytidine
83	9.360	(M+H)^+^	249.1484	250.1556	C_13_H_19_N_3_O_2_	93.27	−2.74	Desethyl-N -acetylprocainamide
84	8.511	(M+H)^+^	308.1534	309.1601	C_19_H_20_N_2_O_2_	86.91	−3.03	DMXB-A
85	5.307	(M+H)^+^	115.0636	116.0709	C_5_H_9_NO_2_	92.37	−2.15	D-Proline
86	7.816	(M+H)^+^	491.2724	492.2797	C_27_H_41_NO_5_S	86.49	−3.72	Epothilone D
87	6.928	(M+H)^+^	204.0900	205.0974	C_11_H_12_N_2_O_2_	83.85	−0.71	Ethotoin
88	8.746	(M+NH_4_)^+^	261.1004	279.1341	C_14_H_15_NO_4_	85.03	−0.98	Ethyl 1-benzyl-3-hydroxy- 2-oxo[5H]pyrrole-4-carboxylate
89	9.297	(M+NH_4_)^+^	373.1958	391.2294	C_15_H_27_N_5_O_6_	83.01	0.87	Gln Asn Ile
90	6.351	(M+H)^+^	300.1433	301.1505	C_12_H_20_N_4_O_5_	99.12	0.11	Gln Gly Pro
91	7.242	(M+H)^+^	406.2211	407.2284	C_20_H_30_N_4_O_5_	81.92	1.31	Gln Leu Phe
92	9.361	(M+H)^+^	421.2328	422.2402	C_20_H_31_N_5_O_5_	96.22	−0.75	Gln Phe Lys
93	7.815	(M+H)^+^	356.2061	357.2133	C_16_H_28_N_4_O_5_	98.89	−0.50	Gln Pro Leu
94	7.150	(M+Na)^+^	340.1749	363.1642	C_15_H_24_N_4_O_5_	84.85	−0.70	Gln Pro Pro
95	6.983	(M+NH_4_)^+^	462.1757	480.2096	C_21_H_26_N_4_O_8_	81.05	−1.42	Glu Trp Glu
96	7.169	(M+H)^+^	172.0854	173.0927	C_7_H_12_N_2_O_3_	97.76	−3.55	Gly Pro
97	8.482	(M+NH_4_)^+^	311.1121	329.1458	C_13_H_17_N_3_O_6_	82.80	−1.26	Gly-Lys-OH
98	14.553	(M+H)^+^	273.2672	274.2746	C_16_H_35_NO_2_	95.51	−1.71	Hexadecasphinganine
99	18.610	(M+H)^+^	101.1200	102.1273	C_6_H_15_N	84.30	3.97	Hexylamine*
100	5.759	(M+H)^+^	226.1065	227.1137	C_9_H_14_N_4_O_3_	99.39	0.42	His Ala
101	8.072	(M+H)^+^	302.1386	303.1461	C_15_H_18_N_4_O_3_	82.69	−2.51	His Phe
102	7.651	(M+H)^+^	417.2011	418.2082	C_20_H_27_N_5_O_5_	96.58	0.17	His Tyr Val
103	7.209	(M+NH_4_)^+^	348.1060	366.1397	C_15_H_16_N_4_O_6_	94.60	2.81	His-Ala-OH
104	8.180	(M+H)^+^	302.1382	303.1453	C_15_H_18_N_4_O_3_	84.08	−1.15	Histidinyl-Phenylalanine
105	5.675	(M+NH_4_)^+^	254.1383	272.1722	C_11_H_18_N_4_O_3_	98.61	−1.64	Histidinyl-Valine
106	6.641	(M+H)^+^	259.1168	260.1239	C_10_H_17_N_3_O_5_	83.20	0.08	Hydroxypropyl-Gamma-glutamate
107	7.566	(M+NH_4_)^+^	268.1173	286.1510	C_11_H_16_N_4_O_4_	81.20	−0.36	Hydroxypropyl-Histidine
108	5.700	(M+Na)^+^	289.1639	312.1534	C_12_H_23_N_3_O_5_	81.82	−0.36	Ile Ala Ser
109	7.815	(M+H)^+^	259.1538	260.1611	C_11_H_21_N_3_O_4_	98.35	−2.20	Ile Gln
110	8.447	(M+H)^+^	356.2061	357.2134	C_16_H_28_N_4_O_5_	94.09	−0.48	Ile Gln Pro
111	6.481	(M+NH_4_)^+^	180.0538	198.0876	C_8_H_8_N_2_O_3_	85.24	−1.88	Isonicotinylglycine
112	18.618	(M+H)^+^	298.1545	299.1616	C_15_H_18_N_6_O	87.32	−0.95	Iso-Olomoucine*
113	8.688	(M+NH_4_)^+^	268.1315	286.1653	C_14_H_20_O_5_	95.31	−1.49	Kamahine C*
114	3.088	(M+NH_4_)^+^	151.0606	169.0945	C_5_H_11_O_5_	83.97	0.54	L-(+)-Arabinose
115	9.360	(M+H)^+^	210.1373	211.1445	C_11_H_18_N_2_O_2_	97.17	−2.43	L,L-Cyclo(leucylprolyl)
116	3.379	(M+Na)^+^	383.1427	406.1318	C_14_H_25_NO_11_	82.74	0.16	Lacto-N-biose I*
117	6.715	(M+H)^+^	196.1218	197.1292	C_10_H_16_N_2_O_2_	81.16	−3.27	L-alpha-Amino-1H -pyrrole-1-hexanoic acid
118	9.355	(M+H)^+^	372.2377	373.2447	C_17_H_32_N_4_O_5_	84.53	−1.09	Leu Ile Gln
119	16.538	(M+H)^+^	195.0538	196.0610	C_9_H_9_NO_4_	83.37	−3.51	Leucodopachrome
120	6.993	(M+H)^+^	259.1905	260.1976	C_12_H_25_N_3_O_3_	80.87	−3.52	Leucyl-Lysine
121	8.258	(M+H)^+^	280.1065	281.1142	C_14_H_12_N_6_O	81.98	2.63	Levosimendan
122	8.072	(M+H)^+^	587.3072	588.3144	C_32_H_45_NO_9_	84.74	3.76	Lipomycin
123	9.360	(M+H)^+^	252.1846	253.1918	C_14_H_24_N_2_O_2_	96.56	−3.25	Lupanyl Acid
124	7.815	(M+NH_4_)^+^	396.2010	414.2348	C_18_H_28_N_4_O_6_	99.69	−0.28	Lys Ser Tyr
125	6.826	(M+H)^+^	309.1685	310.1762	C_15_H_23_N_3_O_4_	87.90	1.22	Lys Tyr
126	4.413	(M+H)^+^	233.1375	234.1451	C_9_H_19_N_3_O_4_	93.49	0.10	Lysinoalanine
127	7.814	(M+NH_4_)^+^	293.1746	311.2084	C_15_H_23_N_3_O_3_	98.62	−2.09	Lysyl-Phenylalanine
128	7.162	(M+H)^+^	309.1693	310.1766	C_15_H_23_N_3_O_4_	98.16	−1.39	Lysyl-Tyrosine
129	8.464	(M+NH_4_)^+^	109.0530	127.0869	C_6_H_7_NO	84.94	−2.26	m-Aminophenol
130	3.516	(M+H)^+^	114.0432	115.0504	C_4_H_6_N_2_O_2_	84.07	−2.51	Muscimol
131	8.440	(M+H)^+^	517.2874	518.2948	C_25_H_43_NO_10_	96.46	2.44	Mycalamide B
132	7.630	(M+H)^+^	203.0800	204.0871	C_8_H_13_NO_5_	95.68	−2.92	N2-Acetyl-L-aminoadipate
133	7.512	(M+H)^+^	130.1109	131.1183	C_6_H_14_N_2_O	86.27	−2.52	N-Acetylputrescine
134	6.990	(M+Na)^+^	175.0989	198.0882	C_11_H_13_NO	82.86	4.87	N-Acetyltranylcypromine
135	7.539	(M+H)^+^	216.1268	217.1341	C_13_H_16_N_2_O	85.32	−2.70	Nb-Acetyl-Nb-methyltryptamine
136	8.747	(M+H)^+^	135.0686	136.0759	C8 H9 N O	87.70	−1.39	N-Benzylformamide
137	9.361	(M+NH_4_)^+^	242.1275	260.1613	C_11_H_18_N_2_O_4_	97.30	−3.29	N-Hydroxypentobarbital
138	19.799	(M+Na)^+^	484.3385	507.3275	C_23_H_44_N_6_O_5_	96.28	−2.34	N-tert-Butyloxycarbonyl-deacetyl-leupeptin
139	19.253	(M+H)^+^	129.1519	130.1592	C_8_H_19_N	99.07	−1.35	Octylamine*
140	15.006	(M+H)^+^	255.2568	256.2641	C_16_H_33_NO	95.59	−2.40	Palmitic amide*
141	9.281	(M+H)^+^	135.0795	136.0868	C_7_H_9_N_3_	94.18	0.93	p-Aminobenzamidine
142	10.483	(M+H)^+^	434.2643	435.2716	C_21_H_34_N_6_O_4_	92.06	−0.34	Phe Arg Leu
143	8.862	(M+NH_4_)^+^	321.1695	339.2035	C_16_H_23_N_3_O_4_	92.71	−2.04	Phe Gly Val
144	9.361	(M+NH_4_)^+^	406.2583	424.2921	C_21_H_34_N_4_O_4_	99.62	−0.75	Phe Lys Leu
145	12.812	(M+H)^+^	243.1991	244.2065	C_17_H_25_N	85.35	−1.83	Phencyclidine
146	18.627	(M+H)^+^	123.9925	124.9998	C_2_H_5_O_4_P	99.77	0.25	Phosphonoacetaldehyde
147	7.154	(M+Na)^+^	542.2482	565.2379	C_23_H_43_O_12_P	83.84	1.95	PI(14:1(9Z)/0:0)
148	8.073	(M+H)^+^	245.1633	246.1708	C_13_H_19_N_5_	80.56	3.12	Pinacidil
149	7.633	(M+NH_4_)^+^	256.0588	274.0926	C_11_H_12_O_7_	98.80	−2.01	Piscidic Acid
150	9.364	(M+Na)^+^	162.1400	185.1293	C_12_H_18_	84.10	4.97	Pregeijerene
151	7.517	(M+H)^+^	186.1008	187.1084	C_8_H_14_N_2_O_3_	84.73	−1.94	Pro Ala
152	6.991	(M+H)^+^	326.1598	327.1669	C_14_H_22_N_4_O_5_	90.54	−2.52	Pro Asn Pro
153	6.934	(M+H)^+^	371.2166	372.2239	C_16_H_29_N_5_O_5_	83.53	0.79	Pro Gln Lys
154	7.164	(M+H)^+^	340.1752	341.1822	C_15_H_24_N_4_O_5_	92.07	−1.60	Pro Gln Pro
155	7.402	(M+H)^+^	269.1378	270.1451	C_12_H_19_N_3_O_4_	99.48	−1.05	Pro Gly Pro
156	7.129	(M+H)^+^	212.1165	213.1239	C_10_H_16_N_2_O_3_	83.55	−2.05	Pro Pro
157	6.930	(M+H)^+^	269.1372	270.1446	C_12_H_19_N_3_O_4_	84.07	1.16	Pro Pro Gly
158	8.222	(M+H)^+^	375.1797	376.1867	C_19_H_25_N_3_O_5_	90.59	−0.64	Pro Pro Tyr
159	5.570	(M+H)^+^	434.2276	435.2349	C_20_H_30_N_6_O_5_	94.18	0.48	Pro Tyr Arg
160	7.417	(M+H)^+^	375.1785	376.1861	C_19_H_25_N_3_O_5_	93.97	2.32	Pro Tyr Pro
161	8.854	(M+Na)^+^	217.1824	240.1717	C_15_H_23_N	85.69	2.86	Prolintane
162	7.820	(M+H)^+^	253.1067	254.1140	C_11_H_15_N_3_O_4_	85.96	−1.67	Pyricarbate
163	5.311	(M+NH_4_)^+^	183.0901	201.1237	C_9_H_13_NO_3_	90.47	−3.06	Racepinephrine
164	7.516	(M+H)^+^	207.0900	208.0972	C_11_H_13_NO_3_	85.18	−2.13	Rhexifoline
165	8.534	(M+H)^+^	244.1584	245.1653	C_15_H_20_N_2_O	88.37	−3.31	Rhombifoline
166	9.862	(M+H)^+^	122.1099	123.1172	C_9_H_14_	86.97	−3.18	Santene*
167	14.655	(M+H)^+^	299.2829	300.2901	C_18_H_37_NO_2_	95.29	−1.68	Sphingosine
168	19.924	(M+H)^+^	213.2457	214.2530	C_14_H_31_N	99.64	−0.40	Tetradecylamine*
169	19.831	(M+H)^+^	370.1547	371.1620	C_21_H_26_N_2_S_2_	93.67	−2.66	Thioridazine
170	4.410	(M+H)^+^	346.2212	347.2284	C_15_H_30_N_4_O_5_	80.69	1.11	Thr Val Lys
171	7.568	(M+Na)^+^	493.3240	516.3131	C_28_H_47_NO_4_S	87.00	−2.91	Tiamulin
172	7.166	(M+H)^+^	253.1068	254.1142	C_12_H_11_N_7_	83.69	3.12	Triamterene
173	6.352	(M+NH_4_)^+^	184.0489	202.0827	C_7_H_8_N_2_O_4_	98.31	−2.85	Trimidox
174	9.211	(M+H)^+^	141.1154	142.1228	C_8_H_15_NO	84.62	−0.41	Tropine
175	7.102	(M+H)^+^	415.1856	416.1930	C_20_H_25_N_5_O_5_	83.43	−0.12	Trp Asn Pro
176	7.010	(M+Na)^+^	418.1850	441.1747	C_20_H_26_N_4_O_6_	80.39	0.59	Trp Asp Val
177	7.210	(M+Na)^+^	372.1803	395.1694	C_19_H_24_N_4_O_4_	81.48	−1.36	Trp Pro Ala
178	7.817	(M+NH_4_)^+^	303.1583	321.1923	C_16_H_21_N_3_O_3_	96.76	−0.18	Tryptophyl-Valine
179	8.506	(M+NH_4_)^+^	423.2000	441.2342	C_20_H_29_N_3_O_7_	88.19	1.20	Tyr Ile Glu
180	8.450	(M+NH_4_)^+^	396.2002	414.2345	C_18_H_28_N_4_O_6_	90.22	1.70	Tyr Ser Lys
181	6.721	(M+Na)^+^	516.2543	539.2431	C_25_H_36_N_6_O_4_S	80.47	−4.67	Udenafil
182	7.666	(M+NH_4_)^+^	387.2258	405.2597	C_20_H_29_N_5_O_3_	80.07	3.07	Urapidil
183	6.933	(M+H)^+^	346.1489	347.1562	C_13_H_22_N_4_O_7_	81.66	−0.18	Val Asp Asn
184	7.580	(M+H)^+^	401.2058	402.2131	C_20_H_27_N_5_O_4_	82.27	1.29	Val His Phe
185	7.655	(M+H)^+^	466.2214	467.2286	C_25_H_30_N_4_O_5_	93.34	0.48	Val Trp Tyr
186	7.189	(M+H)^+^	254.1385	255.1458	C_11_H_18_N_4_O_3_	98.02	−2.38	Valyl-Histidine
187	9.275	(M+NH_4_)^+^	202.0745	220.1084	C_11_H_10_N_2_O_2_	86.30	−1.26	Vasicinone
188	8.958	(M+H)^+^	199.1326	200.1399	C_9_H_17_N_3_O_2_	82.59	−2.81	Vinyl-L-NIO
189	5.660	(M+NH_4_)^+^	157.0856	175.1194	C_6_H_11_N_3_O_2_	98.32	−3.12	V-PYRRO/NO
190	15.142	(M+H)^+^	229.2410	230.2483	C_14_H_31_NO	84.30	−1.90	Xestoaminol C*
191	17.087	(M+H)^+^	115.0461	116.0534	C_5_H_9_NS	94.29	−4.73	xi-2,5-Dihydro-2,4-dimethylthiazole
192	10.173	(M+H)^+^	374.0342	375.0411	C_17_H_12_C_l2_N_4_O_2_	82.26	−1.30	α,4-Dihydroxytriazolam
193	14.599	(M+NH_4_)^+^	222.1991	240.2329	C_15_H_26_O	97.50	−3.28	β-Caryophyllene Alcohol*

* Indicate the metabolites that found similarly from pHMG extract under second evaluation screening. RT=Retention time, DB=Database, LC-MS=Liquid chromatography-mass spectrometry, QTOF=Quadrupole time-of-flight

**Table-4:** Information of the metabolites in pHMG extract. The metabolites are identified by QTOF LC-MS with second screening evaluation.

Number	RT (min)	Ion	Mass	m/z	Molecular formula	Score	DB differences (ppm)	Putatively identified metabolites
1	19.793	(M+Na)^+^	484.3396	507.3290	C_27_H_48_O_7_	96.12	0.87	(25S)-5alpha-cholestan-3beta,4beta,6alpha,8beta,15alpha,16beta,26-heptol
2	3.780	(M+H)^+^	99.0322	100.0394	C_4_H_5_NO_2_	86.24	−1.50	(R)-Dihydromaleimide
3	14.655	(M+NH_4_)^+^	168.1881	186.2219	C_12_H_24_	98.94	−2.06	1-Dodecene*
4	19.883	(M+H)^+^	241.2768	242.2840	C_16_H_35_N	86.03	0.67	1-Hexadecylamine
5	17.082	(M+H)^+^	203.0807	204.0878	C_9_H_17_NS_2_	93.79	−2.07	1-Isothiocyanato-7-(methylthio)heptane*
6	15.586	(M+H)^+^	115.0457	116.0530	C_5_H_9_NS	99.79	−1.42	1-Isothiocyanatobutane*
7	19.896	(M+NH_4_)^+^	392.4383	410.4722	C_28_H_56_	98.76	−0.37	1-Octacosene*
8	17.084	(M+H)^+^	171.1085	172.1157	C_9_H_17_NS	96.61	−1.92	2,5-Dihydro-4,5-dimethyl-2-(1-methylpropyl)thiazole*
9	18.737	(M+H)^+^	170.1303	171.1375	C_10_H_18_O_2_	84.98	2.15	2,6-Dimethyl-3,7-octadiene-2,6-diol
10	7.676	(M+NH_4_)^+^	256.1315	274.1653	C_13_H_20_O_5_	98.83	−1.55	2-[4-(3-Hydroxypropyl)-2-methoxyphenoxy]-1,3-propanediol
11	7.678	(M+H)^+^	113.0845	114.0918	C_6_H_11_NO	98.61	−3.80	2-Acetylpyrrolidine*
12	14.532	(M+H)^+^	105.0790	106.0863	C_4_H_11_NO_2_	84.69	−0.41	2-Amino-2-methyl-1,3-propanediol
13	9.461	(M+H)^+^	144.0422	145.0493	C_6_H_8_O_4_	95.91	0.31	2-Hydroxy-2-(hydroxymethyl)-2H-pyran-3(6H)-one
14	9.859	(M+H)^+^	101.0844	102.0916	C_5_H_11_NO	96.89	−3.15	2-methylbutanal oxime
15	8.921	(M+H)^+^	209.1423	210.1496	C_12_H_19_NO_2_	84.38	−3.50	3,4-dimethoxymethamphetamine
16	19.503	(M+NH_4_)^+^	135.0564	153.0902	C_7_H_7_N_2_O	82.59	−4.21	4-(Hydroxymethyl)benzenediazonium(1+)
17	7.839	(M+H)^+^	125.0839	126.0912	C_7_H_11_NO	87.69	1.30	4-Ethyl-2,5-dimethyloxazole
18	6.992	(M+H)^+^	129.0430	130.0503	C_5_H_7_NO_3_	82.11	−3.43	4-Oxoproline*
19	8.905	(M+H)^+^	153.1156	154.1227	C_9_H_15_NO	95.68	−1.34	5-Butyl-2-ethyloxazole
20	7.182	(M+NH_4_)^+^	165.1155	183.1491	C_10_H_15_NO	82.05	−0.72	5-Methyl-2-(1-pyrrolidinyl)-2-cyclopenten-1-one*
21	16.002	(M+NH_4_)^+^	250.2302	268.2640	C_17_H_30_O	85.38	−2.25	9S,10R-Epoxy-3Z,6Z-octadecadiene*
22	19.912	(M+NH_4_)^+^	364.4070	382.4409	C_26_H_52_	99.67	−0.34	9Z-Hexacosene*
23	5.021	(M+H)^+^	135.0550	136.0622	C_5_H_5_N_5_	86.53	−3.53	Adenine*
24	5.761	(M+H)^+^	226.1066	227.1139	C_9_H_14_N_4_O_3_	92.45	0.04	Alanyl-Histidine*
25	18.616	(M+H)^+^	142.0013	143.0084	C_4_H_2_N_2_O_4_	80.18	0.76	Alloxan
26	5.765	(M+H)^+^	109.0641	110.0713	C_5_H_7_N_3_	83.73	−1.28	Brunfelsamidine*
27	7.804	(M+NH_4_)^+^	922.4758	940.5092	C_44_H_74_O_20_	95.22	1.67	Capsianoside VI
28	19.762	(M+NH_4_)^+^	747.4789	765.5129	C_38_H_69_NO_13_	85.36	−2.63	Clarithromycin
29	17.082	(M+H)^+^	127.1363	128.1435	C_8_H_17_N	86.72	−1.61	Coniine*
30	19.949	(M+NH_4_)^+^	703.4523	721.4865	C_36_H_65_NO_12_	80.50	−2.29	Erythromycin D
31	13.733	(M+Na)^+^	270.1830	293.1723	C_15_H_26_O_4_	86.19	0.32	Ethylene brassylate
32	19.854	(M+NH_4_)^+^	240.2452	258.2791	C_16_H_32_O	85.17	0.68	hexadeca-9-en-1-ol
33	19.781	(M+H)^+^	101.1201	102.1274	C_6_H_15_N	87.12	3.51	Hexylamine*
34	18.616	(M+H)^+^	298.1537	299.1607	C_15_H_18_N_6_O	83.14	1.86	Iso-Olomoucine*
35	8.676	(M+NH_4_)^+^	268.1312	286.1649	C_14_H_20_O_5_	80.46	−0.63	Kamahine C*
36	3.375	(M+Na)^+^	383.1426	406.1318	C_14_H_25_NO_11_	94.48	0.36	Lacto-N-biose I*
37	19.300	(M+H)^+^	129.1511	130.1585	C_8_H_19_N	83.91	4.67	Octylamine*
38	14.588	(M+H)^+^	255.2565	256.2637	C_16_H_33_NO	98.75	−1.08	Palmitic amide*
39	9.858	(M+H)^+^	122.1096	123.1169	C_9_H_14_	85.95	−0.42	Santene*
40	19.911	(M+H)^+^	213.2457	214.2530	C_14_H_31_N	98.18	−0.32	Tetradecylamine*
41	19.838	(M+H)^+^	229.2406	230.2478	C_14_H_31_NO	97.56	0.01	Xestoaminol C*
42	14.568	(M+NH_4_)^+^	222.1988	240.2326	C_15_H_26_O	98.81	−2.08	β-Caryophyllene Alcohol*

* Indicate the metabolites that found similarly from eHMG extract under second evaluation screening. RT=Retention time, DB=Database, LC-MS=Liquid chromatography-mass spectrometry, QTOF=Quadrupole time-of-flight

Sialic acid is known as the key component of EBN because it is served as the unique quantitative marker for grading the EBN. In this study, sialic acid was identified in the eHMG extraction method with the identity of 2,7-Anhydro-alpha-N-acetylneuraminic acid (Table-3). The result agreed with the previous studies that N-acetylneuraminic acid (NANA) is the predominant form of sialic acid in EBN [33-35]. The detected of sialic acid in eHMG extract has further convinced that eHMG extraction method is more suitable as the ideal extraction method.

The type of metabolites present in eHMG and pHMG extracts (from the second screening) was further categorized into five groups based on the macronutrient classification (Figure-2d). The five groups of macronutrients are comprised oligosaccharides, peptides, lipids, nucleosides, and secondary metabolites. There were 192 and 42 metabolites identified from eHMG and pHMG extracts (Tables-3 and 4), respectively. The differences in the type of metabolites between eHMG and pHMG extracts have further supported the preference of the type of metabolites toward each extraction method. Among the macronutrients, eHMG extraction method can extract mostly secondary metabolites, followed by peptides, oligosaccharides, lipids, and nucleosides (Figure-2d). The primary metabolites obtained from this study support the finding from the previous proximate analysis of EBN, which protein is the highest composition followed by carbohydrates and lipids [2,36,37].

The presence of secondary metabolites could most probably explain the recuperative and therapeutic effects of EBN. The secondary metabolite with the identity of O^2^-vinyl 1-(pyrrolidin-1-yl)diazen-1-ium-1,2-diolate (V-PYRRO/nitric oxide [NO]) was found in eHMG extract (Table-3). This secondary metabolite acts as NO donor and delivers NO specifically after metabolism by cytochrome P450 in hepatocytes without affecting the NO-sensitive tissues as well as systolic blood pressure [38]. The *in vivo* study done by Li *et al*. [39] showed that V-PYRRO/NO is able to protect the hindrance to renal congestion and lipid peroxidation from acetaminophen-induced nephrotoxicity in mice. In addition, V-PYRRO/NO can protect against high-fat diet (HFD)-induced liver steatosis and insulin resistance without affecting the mitochondria biogenesis [40]. Interestingly, Zhang *et al*. [41] showed that EBN could prevent HFD-induced insulin resistance by regulating the transcriptional changes in insulin signaling genes. Hence, the presence of V-PYRRO/NO in EBN may explain the protective effect of EBN against the HFD-induced damages. In short, from this study, it is believed that the study on secondary metabolites profiling in EBN in the future is crucial and not to be neglected.

A polysaccharide with an identity of chondroitin was identified from the first screening of eHMG extract (Table-1), in which the discovery of water-soluble chondroitin is similar to the finding of Nakagawa *et al*. in EBN [42]. Chondroitin is a glycosaminoglycan that acts as a chondroprotective agent for the treatment of OA. OA is the lesion of articular cartilage caused by trauma. Since chondroitin is an essential proteoglycan in cartilage, it acts on OA by stimulates the cartilage repair through enhancing the production of the extracellular matrix of cartilage. Besides, chondroitin helps to maintain the viscosity of the synovial fluid to lubricate the joint and therefore reducing the pain of the patient. Furthermore, chondroitin suppresses the inflammatory cytokines such as interleukin-1β that induce the release of matrix metalloproteinases and aggrecanases which cause the degradation of the cartilage [43,44]. In an *in vitro* study done by Chua *et al*. on the effects of EBN to OA [17], the authors reported that EBN can protect articular cartilage from further deterioration by reducing inflammation and enzymatic lesions process and enhancing the cartilage formation simultaneously. Therefore, the effects of EBN on OA might be contributed by chondroitin.

## Conclusion

There was no single extraction method could provide optimal conditions in extracting all the metabolites from EBN. Therefore, complementary extraction methods should be used in parallel when broader metabolite profiles are required. eHMG extraction method was selected as the ideal extraction method for untargeted profiling the type of polar metabolites in EBN. This is because the number and the type of metabolites detected are the highest in eHMG extracts among the four evaluated extraction methods. Furthermore, the presence of key metabolites of sialic acid has further defined the suitability of eHMG extraction method. Therefore, the findings in this study could offer great potential for enhancement in the industrial EBN extraction process and hence improve the overall EBN yield and bioactivities. Nevertheless, the validation of the structure elucidation and functional assays of interesting metabolites shall be carried out in the future.

## Authors’ Contributions

YML conceived the study design. SRT conducted all the designed experiments, data processing, and analysis. THL contributed to the sample collection and performed the in-house extraction method (eHMG and pHMG) for the study. SRT prepared the manuscript with critical feedback from the coauthors. THL, SKC, and YML supervised the study and provided input and advice in the project. All authors have read and approved the final manuscript.
